# Characterisation at the Bonding Zone between Fly Ash Based Geopolymer Repair Materials (GRM) and Ordinary Portland Cement Concrete (OPCC)

**DOI:** 10.3390/ma14010056

**Published:** 2020-12-24

**Authors:** Warid Wazien Ahmad Zailani, Mohd Mustafa Al Bakri Abdullah, Mohd Fadzil Arshad, Rafiza Abd Razak, Muhammad Faheem Mohd Tahir, Remy Rozainy Mohd Arif Zainol, Marcin Nabialek, Andrei Victor Sandu, Jerzy J. Wysłocki, Katarzyna Błoch

**Affiliations:** 1 Faculty of Civil Engineering, Universiti Teknologi MARA, Shah Alam 40450, Malaysia; fadiil2013@yahoo.com; 2 Center of Excellence Geopolymer and Green Technology (CEGeoGTech), Universiti Malaysia Perlis, Perlis 01000, Malaysia; rafizarazak@unimap.edu.my (R.A.R.); faheem@unimap.edu.my (M.F.M.T.); 3 Faculty of Chemical Engineering Technology, Universiti Malaysia Perlis, Perlis 01000, Malaysia; 4 School of Civil Engineering, Universiti Sains Malaysia, Nibong Tebal, Pulau Pinang 14300, Malaysia; ceremy@usm.my; 5 Department of Physics, Częstochowa University of Technology, 42-200 Częstochowa, Poland; nmarcell@wp.pl (M.N.); wyslocki.jerzy@wip.pcz.pl (J.J.W.); katarzyna.bloch@wip.pcz.pl (K.B.); 6 Faculty of Materials Science and Engineering, Gheorghe Asachi Technical University of Iasi, 700050 Iasi, Romania; sav@tuiasi.ro

**Keywords:** microstructure, fly ash, geopolymer, bonding zone

## Abstract

In recent years, research and development of geopolymers has gained significant interest in the fields of repairs and restoration. This paper investigates the application of a geopolymer as a repair material by implementation of high-calcium fly ash (FA) as a main precursor, activated by a sodium hydroxide and sodium silicate solution. Three methods of concrete substrate surface preparation were cast and patched: as-cast against ordinary Portland cement concrete (OPCC), with drilled holes, wire-brushed, and left as-cast against the OPCC grade 30. This study indicated that FA-based geopolymer repair materials (GRMs) possessed very high bonding strength at early stages and that the behavior was not affected significantly by high surface treatment roughness. In addition, the investigations using scanning electron microscopy (SEM) and energy-dispersive X-ray (EDX) spectroscopy have revealed that the geopolymer repair material became chemically bonded to the OPC concrete substrate, due to the formation of a C–A–S–H gel. Fundamentally, the geopolymer network is composed of tetrahedral anions (SiO_4_)^4−^ and (AlO_4_)^5−^ sharing the oxygen, which requires positive ions such as Na^+^, K^+^, Li^+^, Ca^2+^, Na^+^, Ba^2+^, NH^4+^, and H_3_O^+^. The availability of calcium hydroxide (Ca(OH)_2_) at the surface of the OPCC substrate, which was rich in calcium ions (Ca^2+^), reacted with the geopolymer; this compensated the electron vacancies of the framework cavities at the bonding zone between the GRM and the OPCC substrate.

## 1. Introduction

The structural degradation of existing infrastructure, fabricated using ordinary Portland cement (OPC), requires timely maintenance, including a structural strengthening process, repair, and restoration. In the European Union, common types of deterioration, such as spalling, cracking, and delamination, have affected nearly 84,000 reinforced and prestressed concrete bridges [1]. In the United States, it was reported that 27 percent (%) of highway infrastructure bridges were exposed to similar types of uncontrolled deterioration. In fact, approximately half of the government infrastructure assets in Malaysia were noted for similar condition deterioration issues. Thus, repair and restoration are important topics.

Deterioration in the quality of ordinary Portland cement concrete (OPCC) has been commonly reported as a result of many factors, including sulphate attack in underground structures, action of de-icing sea salt, exposure to damp freezing conditions, and alkali–silica reactions [2]. Additionally, failure to adhere to design requirements occurs; for example, insufficient concrete thickness leads to premature damage and structural failure. In some cases, structural damage has been costly for building structures with unique, beautiful, cultural and historical features that must be preserved.

For these reasons, concrete infrastructure restoration and timely maintenance have been in dire need of urgent attention. Commercial repair products that exhibit good mechanical properties and durability have been available on the market, but these products are expensive. Thus, relatively inexpensive alternative repair materials with comparable properties have been studied and have attracted worldwide interest. Geopolymer is one of these inventions, and the earliest study was based on the alkali-activated cement binder in 1938 [3]. The success of this initial study triggered an exponential increase in interest in studies related to geopolymer materials. Since then, researchers have studied and used geopolymer as a repair material after its laboratory tests, such as the slant shear test, to assess the bonding strength between geopolymer repair materials and OPCC substrate as specified in ASTM C 882 [4].

Previous research by Tanakorn et al. implemented high-calcium fly ash (FA) in combination with calcium carbide residue (CCR) for use as a repair material [5]. The high bonding strength and high performance of this geopolymer repair material (GRM) were reported. Bras et al. mentioned that the development of an injectable grout for concrete repairs and strengthening should have high compressive and bonding strength properties and also a self-levelling behaviour [6]. They also mentioned that the grout should have low shrinkage and present good durability behaviour. Recently, Seon-Ju et al. [7] studied the ability of geopolymer as a grouting agent in an aggressive environment. Zhang et al. [8] mentioned that the application of geopolymers as a sealer can be a more effective technique than the commercialized organic polymers.

From the performance of geopolymer, it has been clearly highlighted that the use of geopolymer is potentially to provide increased stiffness under service conditions, and the high bonding strength of geopolymer produces a significant increase in the ultimate strength of the tested composite elements, when compared with existing repair materials. In addition, with a good, high bonding strength, geopolymer guarantees a long-term service life, which helps to avoid the need for repeated repair work on existing concrete structures.

## 2. Materials and Methods

A geopolymer precursor with a composition-featuring high silica (SiO_2_) and alumina (Al_2_O_3_) content is highly recommended for development into repair materials [9,10]. Class F FA (ASTM C618), sourced from Manjung, Perak, Malaysia, was used as the main precursor, and this was combined with an alkaline activator solution to produce the geopolymer. Sodium hydroxide (NaOH) and sodium silicate (Na_2_SiO_3_) solution were combined to form an alkaline activator; the optimum formulation was published in previous studies [11,12].

Concurrently, concrete substrates were produced by using ordinary Portland cement (OPC) binder. OPC, water, and aggregates were combined to produce an ordinary Portland cement concrete (OPCC), which was used as a substrate. The main ingredients and materials used in this study, with their specific suppliers and sources, are listed in Table 1.

### 2.1. Mix Proportions and Sample Preparation

The FA and alkali activator were mixed homogenously, based on an established mix proportion as reported by Mustafa et al. [11]. The geopolymer was moulded in a steel mould and left at a controlled ambient temperature before being subjected to a bonding test. During the casting of the specimens subjected to the bonding strength test, a steel prism mould was divided into two equal layers, where one half was filled with fresh geopolymer and the other half was filled with the hardened OPCC substrate. The geopolymer samples were covered with vinyl sheets and were tested after different curing times. For each test, the average of five samples was reported.

#### 2.1.1. Mix Proportion and Sample Preparation of GRMs

The combination of the FA and alkali activator solution formed a GRM. The combined sodium hydroxide (NaOH) and sodium silicate (Na_2_SiO_3_) solution was prepared to be used as the alkali activator solution. The silicate modulus and NaO% of the final composition were 1.0% and 9%, respectively. The combination of a sodium silicate (Na_2_SiO_3_) solution and a sodium hydroxide (12 M NaOH) solution was used in a formulation ratio of 2.5:1 to form the activator for the geopolymerization process. The sodium silicate (Na_2_SiO_3_) solution-to-sodium hydroxide (NaOH) solution ratio was maintained constant at 2.5:1 for all of the FA-based geopolymer samples; the FA-to-alkaline activator ratio was maintained at 2:1, as optimized in previous work [12].

Solutions of sodium hydroxide (NaOH) and sodium silicate (Na_2_SiO_3_) were mixed together prior to the start of mixing for all samples. These two solutions were mixed and stirred, until a homogeneous solution was obtained. The alkali activator solution needed to be prepared 24 h prior to the application; then the prepared alkali activator was added into the FA. The mixing process was continued, until a homogenous paste was obtained; an ML 712-4 mechanical mixer, featuring a single-phase induction motor and manufactured by TPG Motors & Drives Corporation, Wanchai, Hong Kong was used for this process. The GRM showed an average flow value of 180 mm and a very rapid setting-time (within a range of 30–120 min). The optimum molarity of 12 M NaOH solution was used to attach the geopolymer to the different surface preparations of the OPCC substrate.

#### 2.1.2. Mix Proportion and Sample Preparation of an OPCC Substrate

Type 1 OPC, local river sand, crushed limestone coarse aggregate with a water/cement ratio of 0.5 were prepared as a concrete substrate. The details of the mix proportions of the OPCC substrate were given in Table 2. By referring to the Faury concrete mix design method, a C30/37 strength class of an OPCC was designed, and this grade was also proposed by a previous study for the preparation of the concrete substrate [13].

The OPCC substrate samples were immersed in water for curing purposes and left for 3 months (90 days) to represent the actual concrete substrate condition of existing buildings. This curing period was believed to give the concrete a complete hydration as per existing concrete substrate in-field practice. At 90 days, a compressive strength test was conducted on a cuboid sample (dimension: 50 mm × 50 mm × 125 mm) and yielded an average compressive strength of 35.5 MPa. Samples were prepared for use as an OPCC substrate, by using the mix proportions given in Table 2 for a slant shear bonding test.

#### 2.1.3. OPCC Substrate with Different Surface Preparations

In repairing concrete structures, the preparation of the substrate surface was considered as one of the most important stages. This was to ensure that the substrate surface would be bonded mechanically with the new repair material. The substrate used for the present research was an OPCC, which consisted of cement, water, coarse aggregates, and fine aggregates as previously described. The design grade for the substrate which represented the strength was grade 30, which was the same grade used in the previous method.

**Figure 1:**
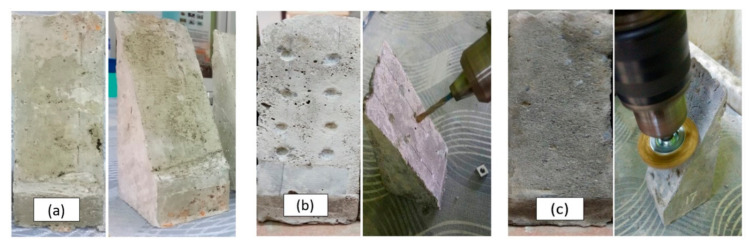
Various types of surface preparation on the OPCC substrate: (**a**) left as-cast; (**b**) drill holes; and (**c**) wire brush.

The samples were cured for 90 days to ensure that the concrete gained sufficient strength. Then, roughening preparations were carried out on the surface of the concrete. Three types of surface textures were adopted and used in these studies which were left as-cast (LA), drill holes (DH), and wire brush (WB). Figure 1 shows the surface preparations of the tested samples.

All of these different types of surface preparation were applied on OPCC specimens, which were used as substrates for the slant shear test. The application of surface treatment onto the hardened concrete required some techniques and procedures. For example, DH were prepared by using drilling machines. WB finishes were prepared by using machines with WB attachments. Finally, no surface treatment was applied to the surface which was labelled as an LA substrate surface. This type of sample was used as a control parameter to analyze the effect of surface treatment on the bonding strength in the absence of any surface preparation.

The objective of having different techniques of surface treatment was to obtain different surface roughness parameters, which was necessary to investigate the effects of each method on the quality of bonding between the geopolymer and concrete substrates. Thus, this facilitated the study of the bond behavior of the GRM with respect to each type of substrate surface treatment and the investigation of the different modes of failure. Based on the highlighted parameters, several tests, conducted in order to measure the properties of geopolymer for the use as a repair material, are described in the next section.

**Figure 2:**
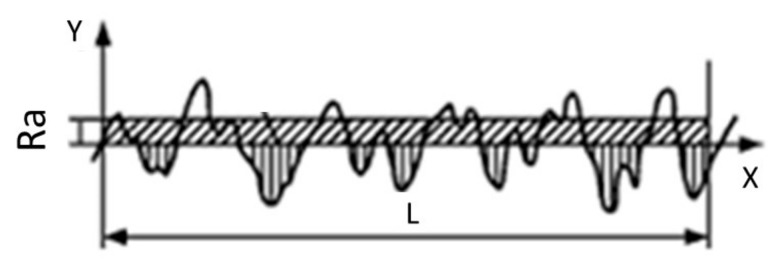
General profile measurement result [14].

Surface preparation is an important aspect of repair and rehabilitation work, because it can help to ensure the effective bonding between the repair material and the substrate. Two surface preparation techniques were implemented on the surface of the concrete substrate, namely, DH and WB. The arithmetic mean deviation of the concrete substrate surface profile was used to measure the roughness, as shown in Figure 2.

Having different kinds of surface preparation enabled different surface roughness parameters to be obtained. In each case, the roughness parameter was measured using a Formtracer CS-3000, Mitutoyo Corporation, Kanagawa, Japan. Roughness was measured by calculating the arithmetic average of the absolute values as shown in Equation (1):

(1) $$\mathrm{Ra}=\frac{1}{L}\int_{0}^{L}\left|f(x)\right|\mathrm{dx},$$

where Ra is absolute roughness; f(x) is the profile height at position x, and L is the evaluation length. A higher value of these deviations indicates a rougher concrete substrate surface.

#### 2.1.4. Bonding Test

The slant shear test of the fresh GRM and hardened OPCC composite was used to evaluate the bonding strength, as described in ASTM C882, using an angle of 30°. This method were used successfully to evaluate the performance of existing inorganic polymer repair materials.

**Figure 3:**
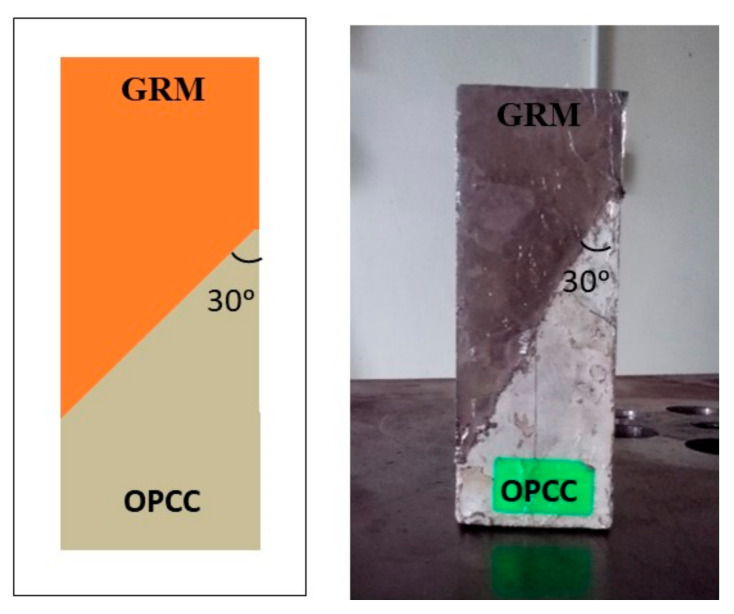
Schematic and experimental diagram of the composite specimen.

The composite specimen consisted of two portions, cast into a 50 mm × 50 mm × 125 mm prism mould. One half of the composite was made up of GRM, and the remaining half was cast with the OPCC substrate. This GRM/OPCC composite substrate, as shown in Figure 3, was used to investigate the strength and performance of each bond between the repair material and the concrete substrate.

**Figure 4:**
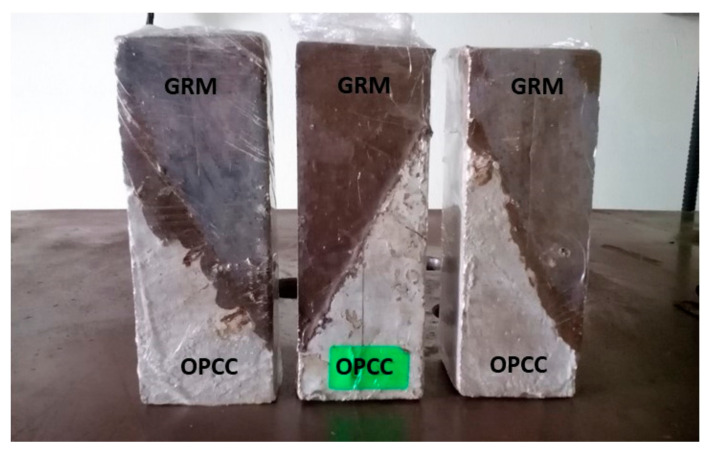
Samples for the slant shear test.

The mixing procedure was started by adding the prepared alkali activator into the FA to form a paste. The fresh geopolymer paste was then poured into the prism mould which were half-filled with the OPCC substrate samples. Each layer was tamped 25 times and vibrated. The samples were left at room temperature (24 ± 2 °C) and wrapped with a vinyl sheet to protect against moisture loss, as shown in Figure 4. 

**Figure 5:**
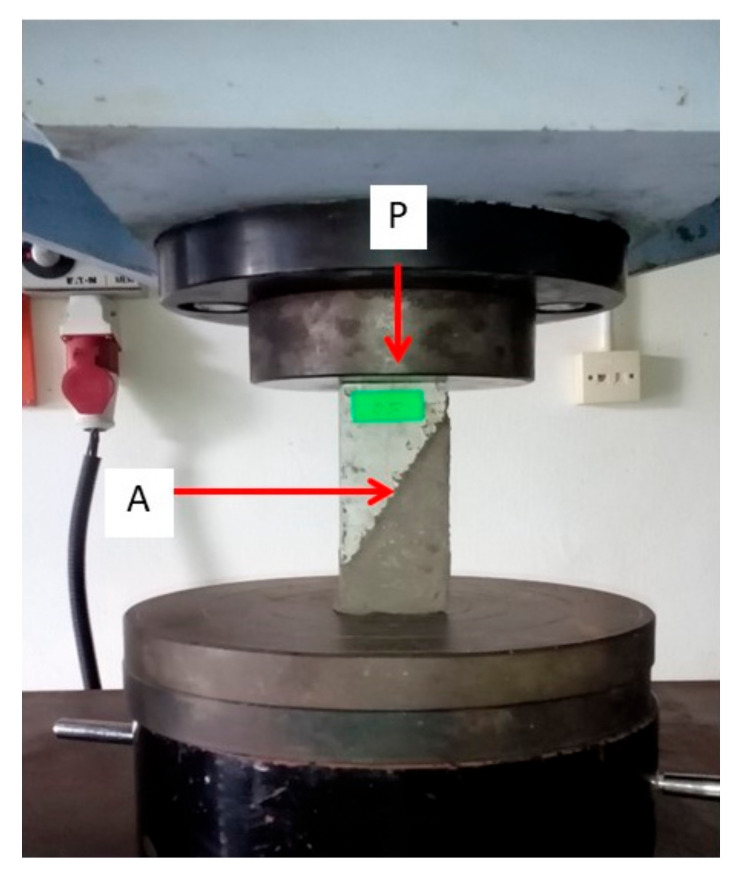
Experimental setup for the slant shear test.

The experiment setup for the slant shear test is presented in Figure 5.

The slant shear specimens were tested at a constant loading rate of 0.50 MPa/s. The mean slant shear bonding strength of five samples was reported as the results of bonding strength between the GRM and the aged OPCC. The slant shear bonding strength was calculated by dividing the maximum load (P) by the bond area (A), as shown in Equation (2):

(2) $$S=\frac{P}{A},$$

where S is the slant shear bonding strength; P is the maximum load; and A is the bond area.

#### 2.1.5. Microstructure of the Interface-Bonding Zone between the GRM and the OPCC

**Figure 6:**
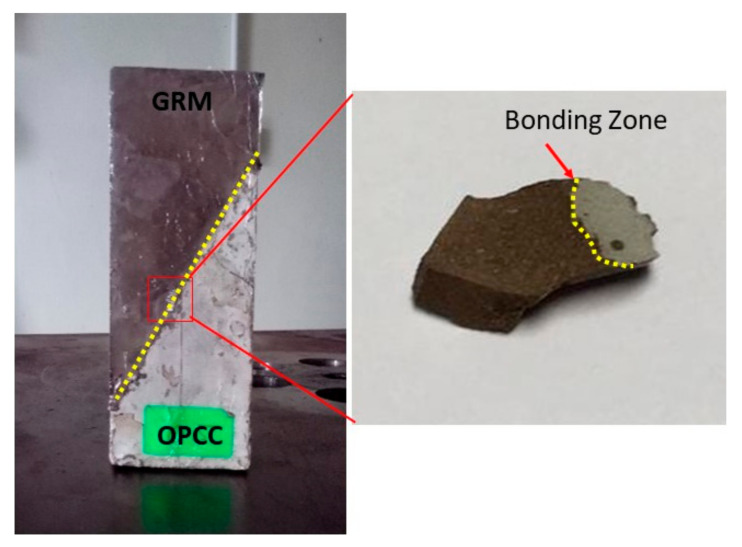
Diagram for the spot area on the sample.

The microstructure images of the FA, FA-based geopolymer, and GRM/OPCC composite substrate were obtained using a JEOL JSM-6460LA Scanning Electron Microscope, JEOL Ltd., Tokyo, Japan which featured secondary electron detectors. The samples for the microstructural analysis of the FA were in the form of powder, as spread onto a carbon tape. In the case of the composite GRM/OPCC specimens, cross-sections were taken by slicing the geopolymer sample (Figure 6). Then, all specimens were coated with palladium by using a JEOL JFC 1600 Auto Fine Coater, JEOL Ltd., Tokyo, Japan before testing. During the imaging process, each surface elemental and compound composition was recorded using energy-dispersive X-ray (EDX) spectroscopy within the same procedure.

## 3. Results and Discussion

### 3.1. Bonding Strength of the GRM, Patched against Different Surface Preparations of the OPCC

**Figure 7:**
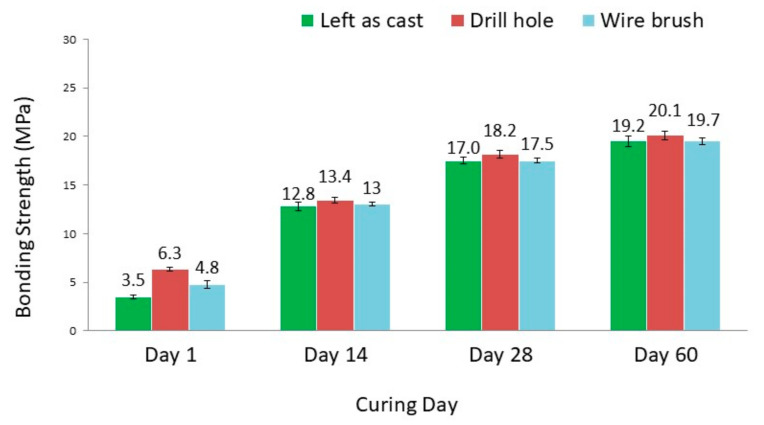
Bonding strengths of geopolymer repair materials (GRMs) with different surface preparations of the OPCC.

Figure 7 shows the overall results of the bonding strengths for different OPCC surface preparation types at different curing ages. 

The maximum bonding strength was obtained for the DH surface after curing for 60 days, i.e., a strength value of 20.1 MPa. Similarly, the highest bonding strengths for LA and WB surface preparations were recorded at the same curing age of 60 days. Furthermore, as curing ages increased, the bonding strength increased significantly especially during the period of 1–14 days. The results indicated that the bonding strength existing within the different surface preparation types between the OPCC and the GRM was enhanced with passing time.

The substrate surface that was LA was considered to serve as a reference, and this surface presented the lowest value of the bonding strength for all curing ages. However, it was evident from the Figure 7 that the highest adhesion values were achieved with the DH surface treatment. Based on the bonding strength, the most effective surface preparation for the concrete repairing purposes was DH.

**Figure 8:**
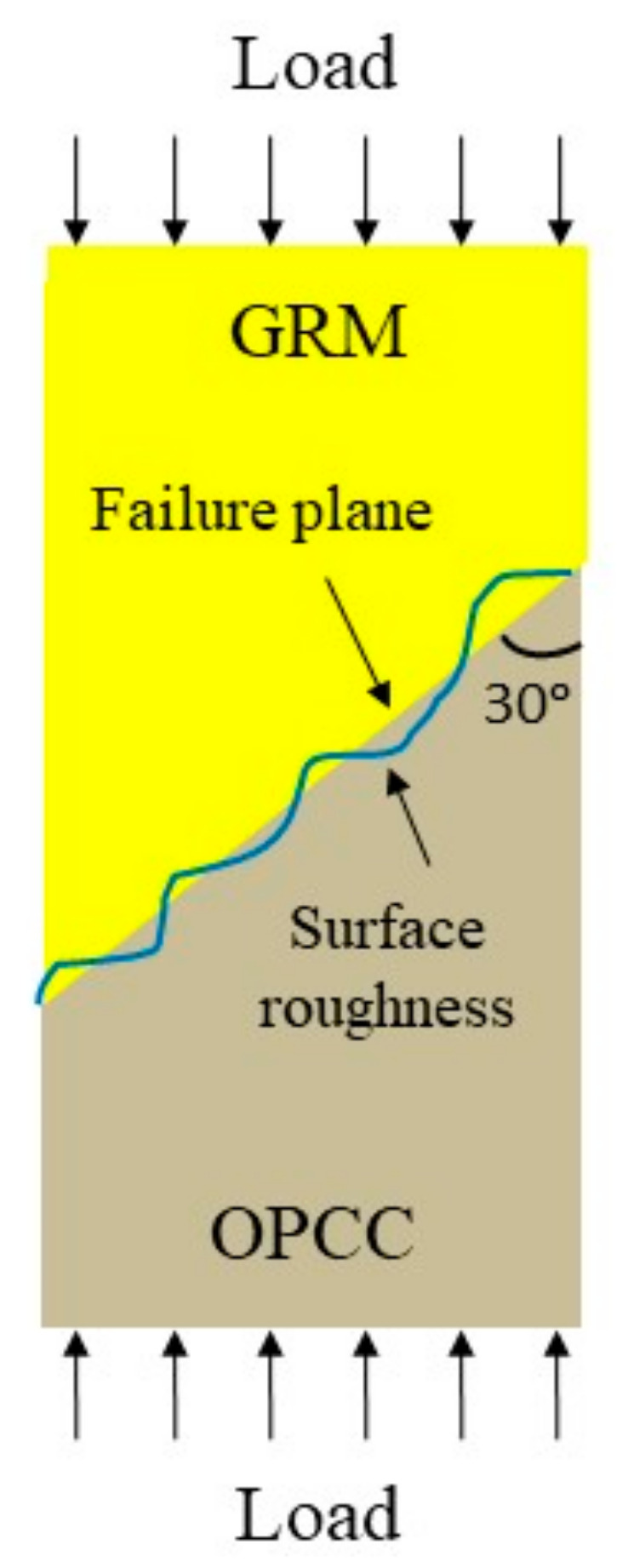
Shear failure at the bonding zone between the GRM and the rough OPCC substrate surface.

When compared to the control reference sample, which was LA, the bonding strength was found to increase in the following order: LA surface < WB surface < DH surface. The bonding strength was improved for different levels of the OPCC surface roughness, as reported in Section 3.3. This bonding strength result suggested that the surface preparation had a bearing and interlocked the bonding, as illustrated in Figure 8.

### 3.2. Failure Mode (Visual Observation) of the GRM/OPCC Composite

**Figure 9:**
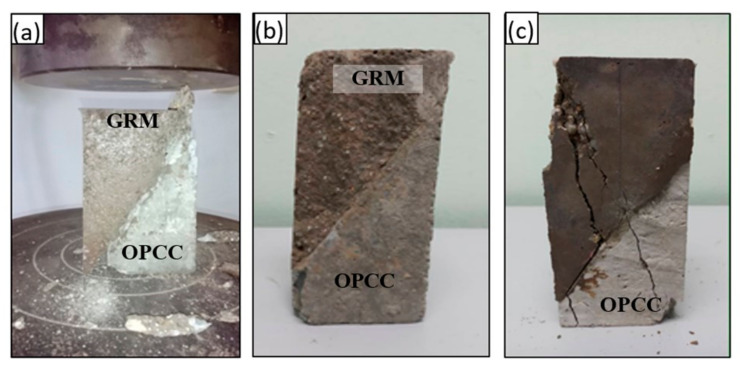
Mode of failure: (**a**) interface failure (Type A); (**b**) substratum failure with good interface (Type B); and (**c**) interface failure and substrate fracture (Type C).

Figure 9 shows three distinct types of failure modes, as observed from the GRM/OPCC overlay specimens after slant shear testing.

The first mode was a pure interfacial failure—wide-ranging de-bonding at the transition zone) where no cracking and rupturing can be observed at both the OPCC and GRM overlay, as shown in Figure 9a. The second mode was an interfacial failure combined with minor concrete substrate cracking or rupture, as shown in Figure 9b. The third mode was a substratum failure with a good interface, as shown in Figure 9c.

These observations indicate that the control substrate (LA) and the WB surface exhibited complete de-bonding at the transition zone failures, as shown in Table 3. The observed trend further emphasized the necessity for appropriate surface preparation to ensure the improved bond strength of composites. The WB surfaces exhibited a combination of cracking at the interface and the substrate, whereas the DH sample exhibited a substrate failure with a good interface.

The failure patterns of the composite samples at later ages were in a monolithic failure mode. The GRM and OPCC acted as one unit, and the crack was formed and ran in a vertical direction, passing through the slant bond area in a similar way to the failure mode of a normal concrete specimen, which indicated excellent bonding. The behavior of this failure showed that the bonding strength between the GRM and the OPCC was developed through chemical bonding and that the strength was developed over time.

The failure patterns of the WB, DH, and LA samples were different at 14 days and 28 days, which were between the combined failure with monolithic failure and adhesive failures. The crack path covered the OPCC, the bonding surface, and the GRM for the sample with WB surface preparation. 

The bonding strength of the GRM with preparation by increasing surface roughness was not significantly different. The results indicated the FA-based GRM did not benefit very significantly through increased surface roughness, in terms of strength. The surface preparations carried out on the OPCC substrate only significantly affected the mode of failure, but not the bonding strength, of the GRM/OPCC composite system.

### 3.3. Surface Roughness Analysis

**Figure 10:**
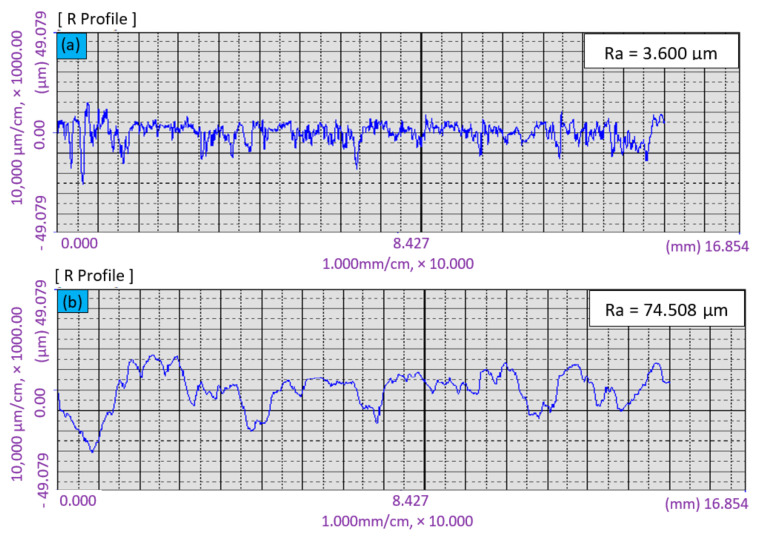
Roughness profiles of substrate: (**a**) left as-cast surface; (**b**) wire brush.

Roughness parameters were determined from the profile of the OPCC substrate surfaces. The profiles of the OPCC substrate specimens for the normal surface (LA) and for the WB surface roughness are presented in Figure 10a and Figure 10b, respectively.

The average values of roughness (Ra) values measured for the normal surface (LA) and WB surface preparation were 3.6 µm and 74.5 µm, respectively. Thus, it was shown that the surface roughness of WB for the OPCC substrate was higher than that of the LA OPCC substrate, which yielded the highest roughness value. In addition, the substrate was made from the OPCC, and thus, the surface preparation of WB generated an aggregate interlocking surface that may improve the bonding strength between the repair materials and the OPCC substrate.

Table 4 demonstrates the relative percentage increases in the slant shear test for WB and DH surface roughness at different ages, as compared to that for the LA surface. The bonding strength increased by varying the surface roughness of the OPCC in a range between 1.5% and 7.0%. The different surfaces preparation provided an average relative increase of 2.5% and 5.5% for WB and DH, respectively. From this obtained result, DH exhibited the highest-bonding-strength increment and improved the bonding strength mechanically.

However, the bonding strengths of the GRM/OPCC composite were almost the same for the LA substrate surface and the other two different surface preparations (WB and DH) for all curing ages. The bonding strength only increased by 2.5% when comparing the bonding strength without surface preparation (LA) and with the (superior) WB preparation.

The bonding that exists between repair material and conventional concrete depends largely on the substrate surface preparation, but not for GRM [13]. In the current study, it was found that the roughness did not affect significantly the bonding strength in the geopolymerisation system, based on the obtained result from the slant shear test, but it did affect significantly the dominant mode of failure.

The bonding strength between the GRM and the OPCC substrate is dependent on the chemical reaction, which is known as the geopolymerisation process at the bonding zone and the interlocking of mineral. However, the scope of studying the bonding between GRM and OPCC discussed in this study focused on the chemical reaction. The geopolymer, used as a repair material, was chemically bonded to the OPCC substrate. Generally, the hardening process via geopolymerization can be divided into three main stages: (1) dissolution of oxide minerals from the source materials (usually silica and alumina) under highly alkaline conditions; (2) orientation of dissolved oxide minerals, followed by coagulation; (3) polycondensation to form a three-dimensional network of the long chain of alumino–silicate structures. The available calcium ions at the surface of the OPCC substrate chemically reacts with dissolved oxide minerals during the second stage of the geopolymerisation process and creates bonding gels which are responsible for the bonding strength between the GRM and the OPCC substrate.

### 3.4. Microstructure Characterization at the Bonding Zone of the GRM/OPCC Composite

**Figure 11:**
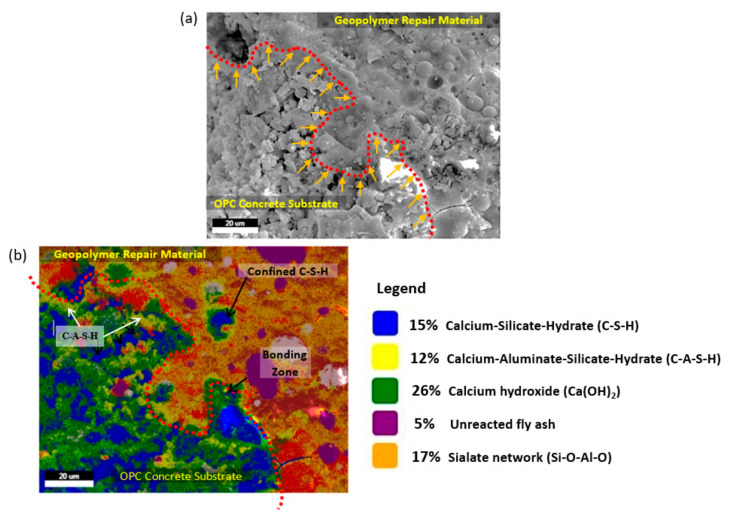
Bonding zone between the GRM and the OPCC substrate: (**a**) microstructure; (**b**) compound element mapping at the bonding zone.

The fracture surfaces arose on the interface areas of the FA-based geopolymer at an optimum NaOH concentration (12 M). The microstructure at the interface area was examined using the secondary and mapping modes of the SEM, as shown in Figure 11.

The micrograph showed the identified interface reaction between the geopolymer and the OPCC substrate.

Figure 11a,b shows the microstructure and compound element mapping at the bonding zone between the GRM and the OPCC substrate with LA surface preparation, respectively. Figure 11a presents the microstructure at the bonding interface between the GRM and the OPCC, where the dotted red line presents a boundary interaction line. At the boundary interaction line, the GRM successfully penetrated the area of the OPCC substrate. The hypothesis was made based on the different densities of materials presented in Figure 11a.

Figure 11b confirms that the GRM penetrated the OPCC zone. In Figure 11b, the element of calcium–aluminate–silicate–hydrate (C–A–S–H) penetrated the OPCC zone. The element of C–S–H occuring in the GRM zone also explained that the penetration of GRM overcame the interfacial bonding zone. From Figure 11b, it is clearly shown that the C–A–S–H gel already penetrated the OPCC substrate. This can be seen from the C-A-S-H gel (yellow coloured) appeared in the OPCC substrate.

During the infiltration of the GRM into the OPCC substrate, the dissolution of calcium hydroxide (Ca(OH)_2_) occurred. Phoo-ngernkham et al. explained the dissolution process of Ca(OH)_2_ by geopolymer [14]. The researcher claimed that geopolymer reacted with Ca(OH)_2_ at the surface of the OPCC produced strength development at the contact zone. The content of calcium ions (Ca^2+^) balanced the aluminium ion, resulting in improving the reaction product at the bonding zone between the GRM and the OPCC substrate. The boundary was identified based on the C–S–H identified in Figure 11b.

Apart from the preparation conditions, an important role was also played by the presence of Ca atoms, which entered the Si–O–Al–O skeleton (i.e., the geopolymer section), and the way, in which they compensated the loading on Al atoms. These loadings are usually compensated by Na^+^ and Ca^2+^ ions. However, they can promote the connection of the individual chain Si–O–Al–O, and it resulted in a structure with a higher strength, given the presence of substances containing Ca. The significant role of Ca atoms in the skeleton of the geopolymer (arising from strength and reduction of leaching) was also shown by the results from the calcium distribution mapped in the Figure 11b.

Detailed explanation of the bonding mechanism was explained by the compound identified from the elemental distribution at the interface. The process of geopolymerization is divided into three main stages, which are dissolution, gelation, and polycondensation. In this work, the dissolution and gelation processes mainly occurred in the GRMs during the fresh state, when FA was dissolved in the optimum alkali solution (12 M) [9]. During repair work, the GRM (in the fresh state) was patched onto the OPCC substrate, and then a new geopolymer structure, identified as a C–A–S–H gel, was developed along the bonding zone as mapped.

## 4. Conclusions

The following conclusions were reached based on the bonding strength and microstructure analysis as presented in this paper:

1. Bonding strength was not affected significantly by high-roughness surface treatment.
2. The bonding strength between the geopolymer and concrete substrate confirmed that geopolymer adhered to and interlocked well with the artificially prepared concrete substrate and consequently enabled a safe and efficient restoration process.
3. The availability of calcium hydroxide (Ca(OH)_2_), which was rich in calcium ions, at the OPCC substrate as a result of the hydration process, played a significant charge-balancing role.

## Figures and Tables

**Table 1:** Overall summary of materials used for the study.

Material	Function	Supplier
Fly ash	Aluminosilicate source	Manjung Powerplant, Perak
Sodium silicate solution	Alkali activator	South Pacific Chemicals Industries Sdn. Bhd, Malaysia
Sodium hydroxide pellet	Alkali activator	Formosa Plastic Corporation, Taiwan.
Type 1 ordinary Portland cement (OPC): 32.5R	Cement used as a concrete substrate	Lion, Alor Setar, Kedah
Aggregate	Filler	CAH Enterprise (P) Sdn. Bhd., Perlis

**Table 2:** Mix proportions and strengths of the OPC.

Components	OPC (kg/m^3^)	Aggregates (kg/m^3^)		Water (kg/m^3^)	Compressive Strength (MPa)
		Fine	Coarse		
OPC concrete (OPCC) grade 30	410	648	1152	205	30.5

Compressive strength = strength cured at 90 days for a specimen (dimension: 50 mm × 50 mm × 125 mm).

**Table 3:** The failure patterns of the GRM/OPCC composite after the slant shear tests.

Ageing	Surface Preparations		
	Left as-Cast	Wire Brushed	Drill Holes
Test results at 1 day	Type A	Type B	Type B
Test results at 14 days	Type A	Type C	Type B
Test results at 28 days	Type A	Type C	Type B
Test results at 60 days	Type C	Type C	Type C

**Table 4:** The relative increase in slant-shear strength for wire brushed and drilled holes surface preparations.

Ageing	Bonding Strength Increment	
	Wire Brushed	Drill Holes
Test results at 1 day	3%	5.7%
Test results at 14 days	1.5%	4.7%
Test results at 28 days	2.9%	7.0%
Test results at 60 days	2.6%	4.7%
Average	2.5%	5.5%
